# POSTN promotes proliferation and epithelial-mesenchymal transition in renal cell carcinoma through ILK/AKT/mTOR pathway

**DOI:** 10.7150/jca.51253

**Published:** 2021-05-13

**Authors:** Yuan-yuan Jia, Yue Yu, Hong-jun Li

**Affiliations:** 1 Department of Health Management Medical Center, China-Japan Union Hospital of Jilin University,126 Xiantai Street,Changchun, Jilin, China.; 2 Department of endocrinology and metabolism, China-Japan Union Hospital of Jilin University, 126 Xiantai Street,Changchun, Jilin,China.

**Keywords:** POSTN, Renal cell carcinoma, epithelial-mesenchymal transition, ILK/AKT/mTOR pathway.

## Abstract

Periostin (POSTN) is an extracellular matrix (ECM) protein, involved in various diseases. This research focused on the detailed mechanisms study of periostin (POSTN) overexpression in renal cell carcinoma (RCC) invasion and migration. Western blot and RT-PCR were performed to explore POSTN expression in various RCC cells. Cells were transfected with siRNAs or lentivirus to regulate the expression of POSTN. The effects of POSTN on cell viability, apoptosis, migration, invasion and epithelial-to-mesenchymal transition (EMT) of RCC cells were determined by CCK-8, flow cytometry, migration and invasion assay and Western blot analysis. POSTN expression was significantly enhanced in RCC cells compared with renal tubular epithelial cells. *In vitro* experiments showed that POSTN knockdown could dramatically inhibit RCC cell proliferation, migration and invasion, while overexpression of POSTN could promote these biological behaviors. We further demonstrated that POSTN knockdown suppressed epithelial-mesenchymal transition (EMT), which was mediated via upregulation of E-cadherin and downregulation of N-cadherin and vimentin, through IKL/AKT/mTOR pathway. In contrast, overexpression of POSTN could promote EMT in RCC cells via the activation of IKL /AKT/mTOR pathway. Next, we demonstrated that higher POSTN expression promoted angiogenesis *in vivo* in an RCC xenograft tumor via activating IKL /AKT/mTOR pathway. Our study showed that POSTN could promote EMT through ILK/AKT/mTOR pathway and might be an alternative therapeutic strategy for RCC treatment.

## Introduction

Renal cell carcinoma (RCC), is one of most deadly malignant cancers around the world, which causes about 10000 deaths every year 1. According to American Cancer Society (ACS), around 65340 were diagnosed with RCC, and 14970 patients were died of RCC in 2018 1. RCC patients often have recurrence and/or metastasis in late stage, which leads to poor clinical results, even though the therapeutic strategy has been developed rapidly 2, 3. Targeted molecular therapy is considered as an efficient method for RCC metastasis treatment, and some targeted molecular, like mTOR inhibitor (temsirolimus) and VEGF (bevacizumab), have been proved to be clinically efficient for RCC treatment 4, 5. Even though several breakthroughs have been achieved in RCC research over past decades, the RCC metastasis mechanism still remains unclear because of the complexity of malignant tumors, which causes the low efficiency and drug-resistance of targeted molecular therapy 6. Many genes related to RCC have been identified, but the heterogeneity of disease increases the importance of identifying new genes and further understanding the molecular mechanism of RCC 7, 8.

Periostin (POSTN) is an extracellular matrix (ECM) protein that participates in the regulation of intercellular adhesion by interacting with other ECM proteins, including fibronectin, tendon protein C, type V collagen and perimyosin 9, 10. In periosteum and periodontal ligament, POSTN is highly expressed in early osteoblasts and has been found to play a role in bone and tooth formation and structural integrity maintenance of these tissues 11. In addition, overexpressed POSTN was thought to be related to various cancers, including colon cancer, lung cancer, pancreatic cancer, and prostate cancer 12-15. Previous research also revealed that POSTN was associated with carcinoma invasion and metastasis 16, which provide a potential target for cancer metastasis treatment. Previous studies suggested that POSTN was involved in RCC cell attachment and differentiation 17, 18. However, the involvement of POSTN in other biological processes such as migration and invasion of RCC is still unclear.

In this study, POSTN was found to be elevated in RCC patients, and the expression of POSTN was detected in several RCC cell lines. Moreover, the effects of POSTN knockdown and overexpression on the proliferation, colony formation and invasion of RCC cells were studied both *in vitro* and *in vivo*. Last, western blot was used to analyze the role of POSTN on ILK/AKT/mTOR pathway.

## Materials and methods

### Patients and tissue samples

A total of 37 RCC tissues, paired with adjacent normal tissues, were obtained from patients who underwent nephrectomy in the China-Japan Union Hospital of Jilin University. All tissue samples were immediately snap frozen in liquid nitrogen after resection and then maintained at - 80°C for further use. The pathologic diagnosis of RCC for these patients was confirmed by senior pathologists. The TNM stages were determined according to the 2010 American Joint Committee on Cancer (AJCC) classification system, and the nuclear grades were assigned in accordance with the Fuhrman nuclear grading system. All experimental procedure was approved by the Ethics Committee of the China-Japan Union Hospital of Jilin University.

### Cell lines and cell culture

The human renal cell carcinoma cell lines A498, Caki-1, ACHN and 786-o were purchased from the American Type Culture Collection (ATCC, Manassas, VA, USA). Normal human primary kidney tubular epithelial (HK-2) cells were obtained from Cell Bank of the Chinese Academy of Sciences (Shanghai, China). All cancer cells were cultured in RPMI 1640 culture media supplement (Invitrogen, Carlsbad, CA, USA) with 10% fetal bovine serum (FBS, Sigma, St. Louis, MO, USA). NHK cells were grown in Dulbecco's Modified Eagle's medium (Invitrogen, Carlsbad, CA, USA) with 10% fetal bovine serum. All the cells were cultured at 37 °C in a humidified atmosphere of 5% CO_2_. OSU-T315 (CAS 1333146-24-9, ILK inhibitor, CA, USA) were prepared in dimethyl sulfoxide (DMSO) and stored at ‑20˚C. A498 and ACHN cells were seeded at 1x10^6^ cells per 60‑mm dish and incubated at 37˚C for 16 h. Inhibitors (0.1% of culture medium) were then added to culture media (OSU-T315, 0.1 µM), and cells were incubated at 37˚C for 48 h.

### Cell viability assays

Cell viability was evaluated using the Cell Counting Kit-8 (CCK-8) (Dojindo, Shanghai, China) in accordance with the manufacturer's instructions. Briefly, cells (1 × 10^4^) were seeded in each well of a 96‐well plate 10 μl CCK-8 solution was added into each well and then incubated at 37 °C, protected from light for 4 h. Optical density was measured at 450 nm. The assay was conducted at 0 h, 24 h, 48 h, 72 h and 96 h to generate a growth curve.

### Colony formation assay

When a single cell proliferates for more than 6 generations *in vitro*, each group of cells in the logarithmic growth phase is digested with 0.25% trypsin and pipetted into single cells, and the cells are suspended in RPMI 1640 culture medium of 10% fetal bovine serum spare. The cell suspension was diluted in multiples of gradients, and each group of cells was inoculated into a dish containing 10 mL of 37°C pre-warmed culture solution at a gradient density of 50, 100, and 200 cells/dish, and gently rotated to evenly disperse the cells. Incubate in a cell incubator at 37°C 5% CO_2_ and saturated humidity for 2 to 3 weeks. It is often observed that when there are visible clones in the petri dish, the culture is terminated. The supernatant was discarded and carefully rinsed twice with PBS. Add 4% paraformaldehyde to fix cells 5mL and fix for 15 minutes. Then remove the fixing solution, add an appropriate amount of GIMSA and apply the staining solution to stain for 10 to 30 minutes, then slowly wash off the staining solution with running water and air-dried. Invert the dish and overlay a transparent film with a grid, directly count the clones with the naked eye, or count the number of clones greater than 10 cells in the microscope (low magnification).

### Wound healing assay

Cells (5×10^5^) were seeded in six-well plates and cultured to produce a confluent monolayer. Wound areas were scraped using 200-μl pipette tips. Washed three times with PBS to remove debris and then adding RPMI 1640 culture media without FBS. Wound closure was observed and photographed at 0-48 h under an inverted microscope.

### Migration and invasion assay

In migration assay, 1 × 10^5^ cells were centrifugated, which were resuspend in 100µl serum-free medium in the upper chamber, then the lower chamber was added with 600µl of medium containing 10% FBS, and incubated at 37°C for 24 h. The culture medium in the upper chamber was discarded, and the lower chamber was added with 600µl of 4% paraformaldehyde for 20 min fixation, then washed with PBS for 1-2 times. 400µl of 0.1% crystal violet was added to the lower chamber and stain for 10 minutes. Migratory cells through the polycarbonate membrane after 6 h, and invasive cells after 48 h were observed invertedly under microscope in at least five randomly selected fields.

### Real‑time quantitative reverse transcription PCR (qRT‑PCR) analysis

Total RNA was isolated from cells or tissue by RNAiso Plus (Takara, Kusatsu, Japan). Reverse transcription was performed before quantification of POSTN and GAPDH, using PrimeScript™ RT reagent Kit (Takara, Kusatsu, Japan). The following primer sets for POSTN were used: POSTN primers forward: 5′- AGGTCACCAAGGTCACCAAA-3′, reverse: 5′- TTCCTCACGGGTGTGTCTCC-3′; GAPDH primers forward: 5′- TCGGAGTCAACGGATTTGGT -3′, reverse: 5′- TTCCCGTTCTCAGCCTTGAC-3′. Relative quantification was performed with the 2-∆∆Ct method.

### Western blot analysis

Total protein was isolated from cells or tissue by 100µl RIPA lysis buffer and separated by polyacrylamide gels, and then transferred onto PVDF membranes. After blocked with TBS-T solution for 1 h, the membranes were incubated with primary anti-bodies, including POSTN (cat. no. sc-398631; 1:500), E-cadherin (sc-8426, 1:1000), vimentin (sc-80975, 1:1000), N-cadherin (sc-8424, 1:1000), ILK (sc-20019, 1:1000), AKT (sc-55523, 1:1000), p-Akt (sc-81,433, 1:1000) and mTOR (sc-517464, 1:1000), and p-mTOR (sc-293133, 1:1000). Membranes were incubated with secondary antibodies for 1 h at room temperature. The western blots were visualized using an enhanced chemiluminescence system (GE Healthcare, Chicago, IL, USA).

### Immunofluorescence

The cells were seeded on coverslips and washed with PBS for 3 times. 4% paraformaldehyde was used to fix cells on coverslips, and the cells were permeabilized with 0.5% Triton X-100 followed by blocking with goat serum for 30 min. Then, the cells were incubated with primary antibody against POSTN at a 1:500 dilution overnight. After incubation with anti-rabbit secondary antibodies (ZSGB-BIO, Beijing, China), the nuclei were counter-stained with 0.2 mg/mL 4′,6-diamidino-2-phenylindol (DAPI). Images were captured by an Olympus confocal microscope.

### IHC staining

Tumor tissue samples were collected from sacrificed mice, fixed in 4% paraformaldehyde in PBS for at least 72 h, and dehydrated in increasing concentrations of ethanol. All samples were then embedded in paraffin. For IHC staining, the tissue was placed on glass slides, rehydrated, incubated with 3% hydrogen peroxide to quench endogenous peroxidase activity, and then blocked by goat serum for 20 min. The tissues were incubated with primary mouse anti-human periostin at 1:500 dilutions, at 4°C overnight. After undergoing three PBS washes, tissues were incubated with secondary antibody. After three washes in Tris buffer for 5 min each, a diaminobenzidine (DAB) substrate kit (Biogenex, San Ramon, CA) was used to visualize the antigen/antibody complex.

### RNA interference and cell transfection

siRNAs targeting POSTN (siRNA: 5′-GCTTGGGACAACTTGGATTCT-3′) was constructed by Genepharma (Suzhou, China). Lipofectamine™ 3000 (Thermo Fisher Scientific, MA, USA) was used for transient transfection. After 48 h transfection, the functions of siRNA were assayed. Two types of lentivirus plasmids LV-POSTN and LV-NC (blank vector), conducted from Sangon Biotech (Shanghai, China) were transfected into RCC cells to establish the overexpressed group and the control group.

### Cell‐apoptosis analysis

Cell apoptosis was assessed via flow cytometry (FCM) analysis. Different cells were harvested for 48 h, then washed with ice‐cold phosphate‐buffered saline (PBS), and stained with annexin V-fluorescein isothiocyanate apoptosis detection kits (BD Biosciences, San Diego, CA). The stained cells were measured using a flow cytometer (BD Biosciences).

### *In vivo* xenograft tumor growth assay

The cells washed with PBS twice, and digested with trypsin, then resuspend the cells with serum-free medium to obtain suspension contains 1×10^7^ cells in 200 μL. Three weeks old nude mice (BALB/c nude mice) were fed for 1 week, and each was subcutaneously inoculated with 1×10^7^/200 μL cells on the right axillary wall. Observe the growth of the tumor daily, and use a vernier caliper to measure the tumor volume after the tumor emerges. Tumor volume = (D× ^2^)/2 (D represents the long diameter of the tumor and d represents the short diameter of the tumor). Observe and measure every 3 days to calculate the relative tumor volume (RTV). The average growth volume of the transplanted tumor in each group of animals was used to draw the growth curve of the transplanted tumor. At the end of the experiment, the nude mice were photographed on the ultra-clean workbench, the tumor was completely peeled off, the size of the tumor was measured with a vernier caliper, the weight of the transplanted tumor was weighed with an electronic balance, the tumor was taken out with surgical scissors, and the picture was taken.

### Statistical analysis

Data are presented as the mean ± s.d. of at least three independent experiments. Statistical analysis was done by the software SPSS 18.0.0. The quantitative* in vitro* and *in vivo* data were analyzed using the Student's t-test (two-tailed). In this study, p-values <0.05 were considered statistically significant.

## Results

### POSTN expression levels in RCC and corresponding normal tissues

**Figure 1:**
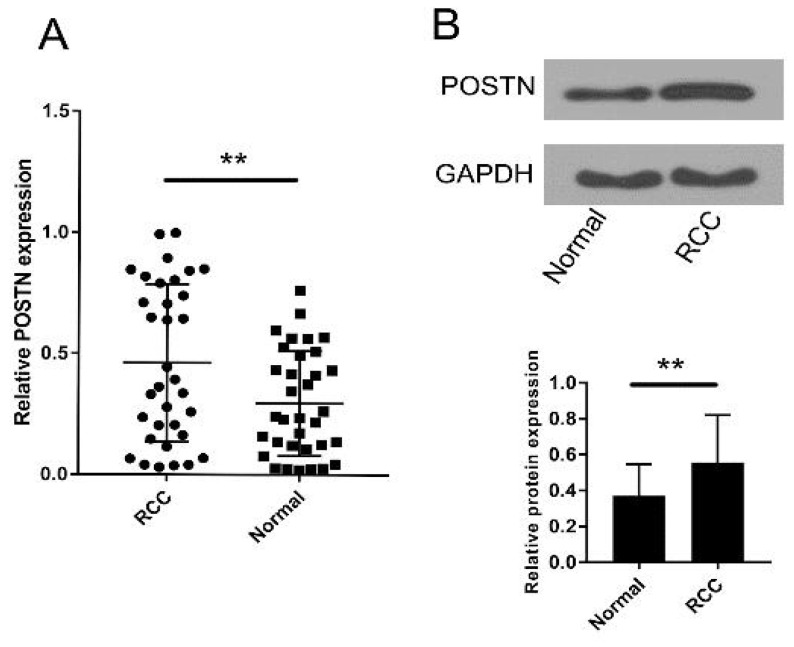
Expression of POSTN in RCC. (A) POSTN expression levels were significantly upregulated in RCC tissues compared to adjacent normal tissues. (b) POSTN protein expression levels were significantly upregulated in RCC tissues compared to adjacent normal tissues. **p<0.01 compared with normal tissues.

To investigate the role of POSTN in RCC, POSTN expression was detected in tumor and corresponding normal tissue specimens from 37 RCC patients and several cell lines by qRT-PCR. As shown in Figure 1A, POSTN was significantly upregulated in RCC tissues compared to adjacent normal tissues (P < 0.01). Then, the western blot was used to analyze the POSTN expression in RCC patients Figure 1B, which is similar to the qRT-PCR result.

### Upregulation of POSTN in renal cell carcinoma cells

**Figure 2:**
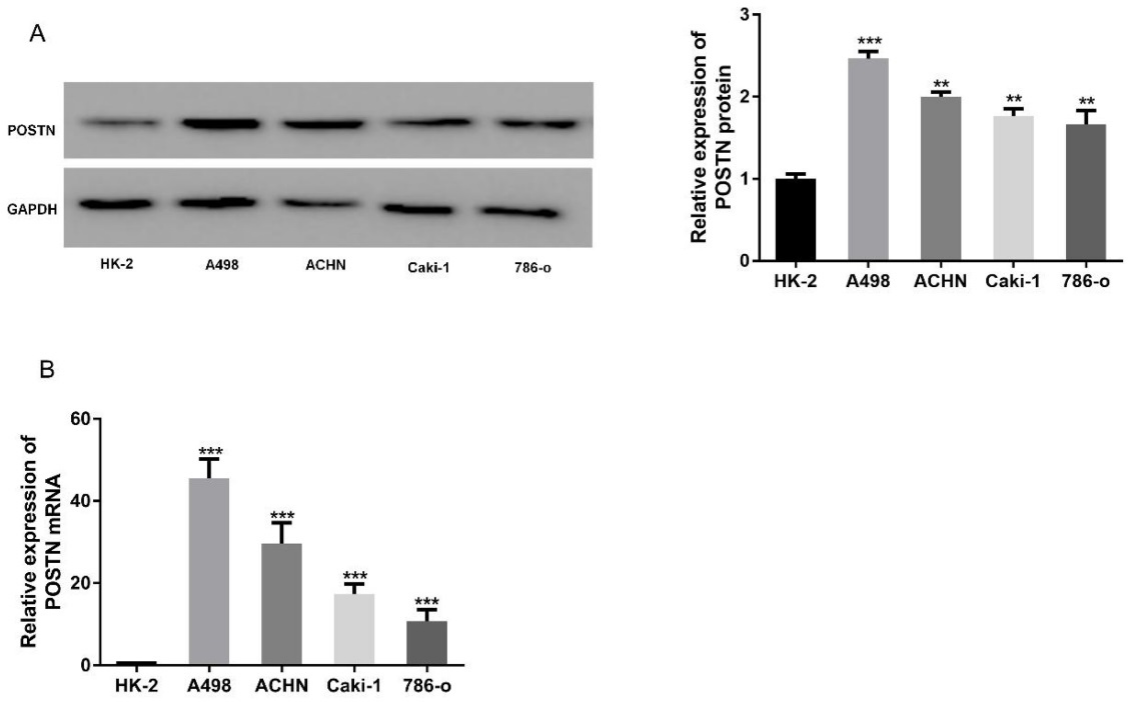
POSTN expression in various RCC cells. (A) Western blot analysis of POSTN; (A) mRNA expressions of POSTN in different cells. Data are presented as means ± s.d. **p<0.01, ***p<0.001 as compared with si-NC and LV-NC groups.

To explore the expression of POSTN in RCC, RCC cells (Caki-2, 786-O, ACHN and A498) were chosen for POSTN analysis, using an immortalized normal renal tubular epithelial cell line (HK-2) as a control. Western blot showed POSTN was overexpressed in RCC cells, compared with HK-2 cells (Figure 2A), which was consistent with the mRNA expressions between different groups (Figure 2B). All these results suggested that the abnormal expression level of POSTN might be correlated with RCC malignancy.

### POSTN‑enhanced cell proliferation and colonization in RCC cells

**Figure 3:**
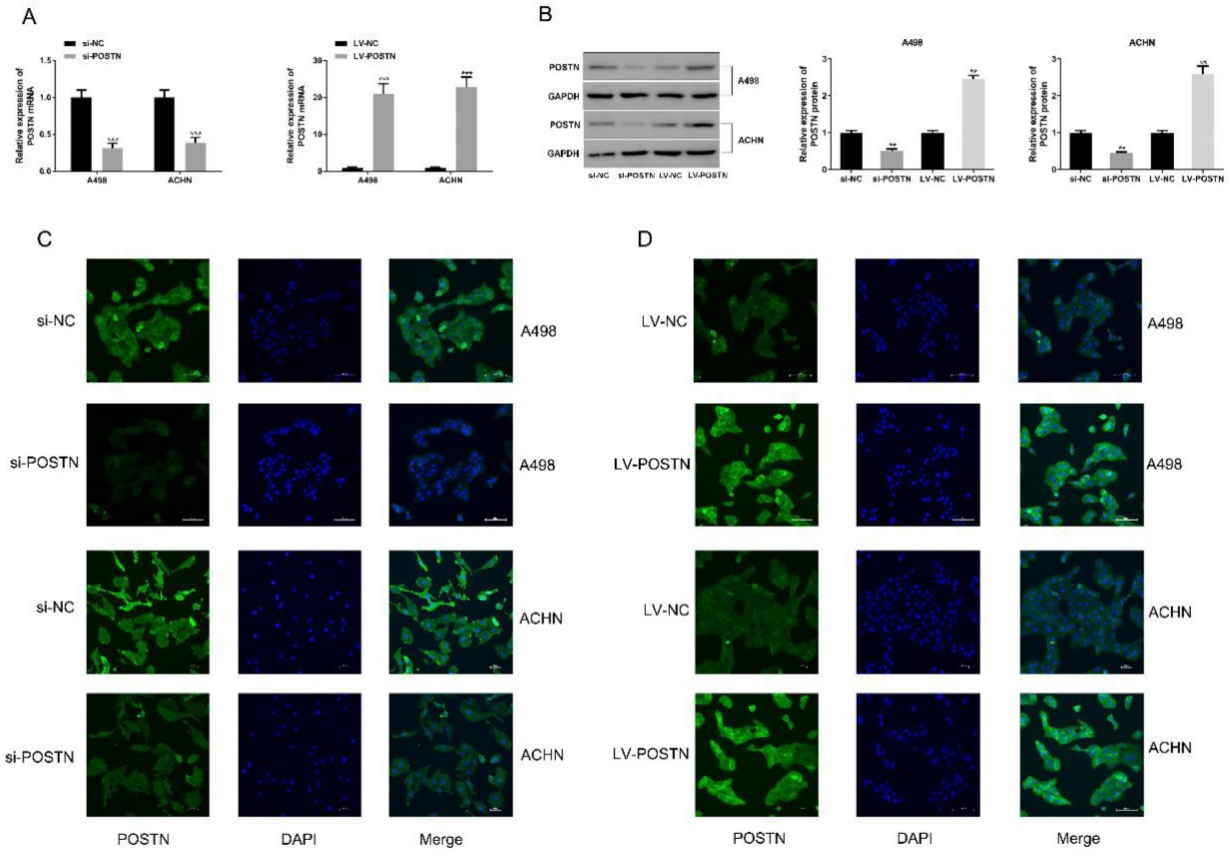
The POSTN expression in RCC cells. (A and B) The RNA and protein expression of POSTN in ACHN and A498 cells were detected; (C and D) Immunofluorescence analysis of POSTN expression in ACHN and A498 cells (Bar: 100 µm).

To study the potential oncogenic role of POSTN in RCC, knockdown studies were performed, using small interference RNA as a method. In both ACHN and A498, POSTN expression levels were effectively reduced with the transfection of si-POSTN (Figure 3A, B). Moreover, immunofluorescence staining was used to observe both POSTN location and expression in RCC cells, showing that si-POSTN could dramatically decrease the POSTN expression in A498 while POSTN expression was still observed after POSTN knockdown in ACHN (Figure 3C). In addition, POSTN was mainly distributed in the cytoplasm of RCC cells, and cell nuclei appeared mostly oval and blue under a light microscope (Figure 3C). Next, cell proliferation was assayed after POSTN knockdown in RCC cells using CCK-8 detection. The result showing that POSTN knockdown could significantly reduce the growth of RCC cells compared with siRNA scramble (Figure 3E, P < 0.05) after 48h incubation. Then, colony formation result showing that knockdown of POSTN significantly inhibited colony formation capacity of ACHN and A498 (Figure 3F).

**Figure 4:**
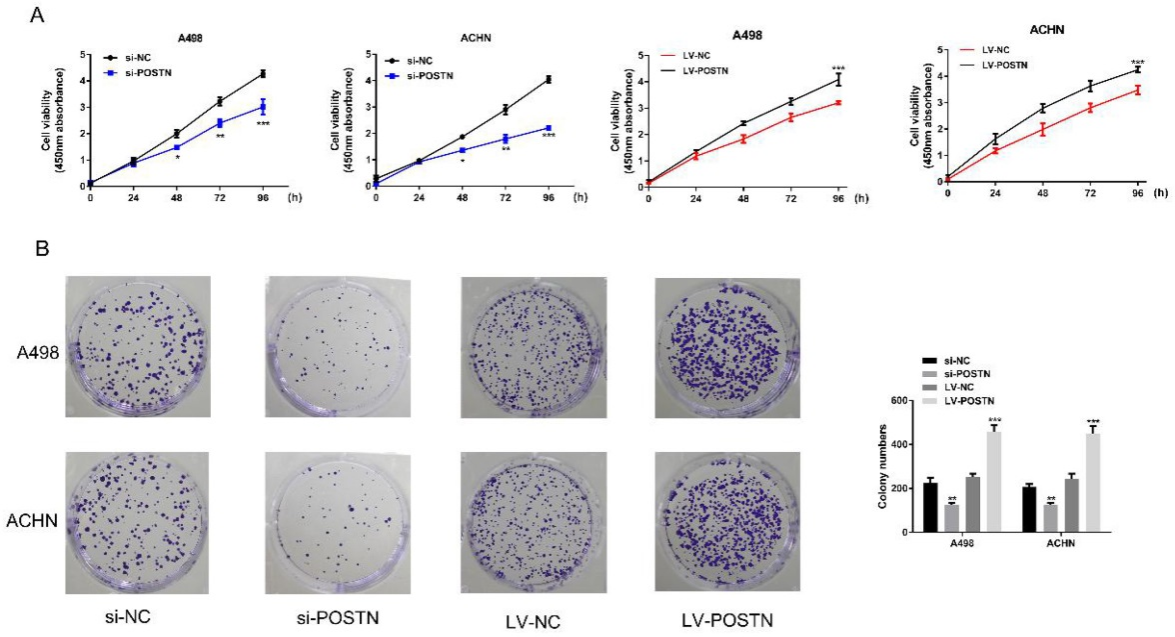
POSTN-enhanced cell proliferation and colonization in RCC cells. (A) ACHN and A498 cells proliferation after POSTN knockdown and overexpression; (B) Colony formation with POSTN knockdown and overexpression in ACHN and A498. All cell assays were performed in triplicate. Data are presented as means ± s.d. *p<0.05, **p<0.01, ***p<0.001 as compared with si-NC and LV-NC groups.

To confirm the effect of POSTN on cell proliferation and colonization in RCC cells, we next transfected POSTN-overexpressed lentivirus (LV-POSTN) into ACHN and A498, and stably increased CEP55 expression levels in RCC cells (Figure 3B, D). Then we found that upregulated POSTN significantly increased colony formation capacity and proliferation of ACHN and A498 (Figure 4A, B). These results suggested that POSTN promoted cell proliferation and colonization capacity in RCC.

### POSTN promotes migration and invasion in RCC cells

**Figure 5:**
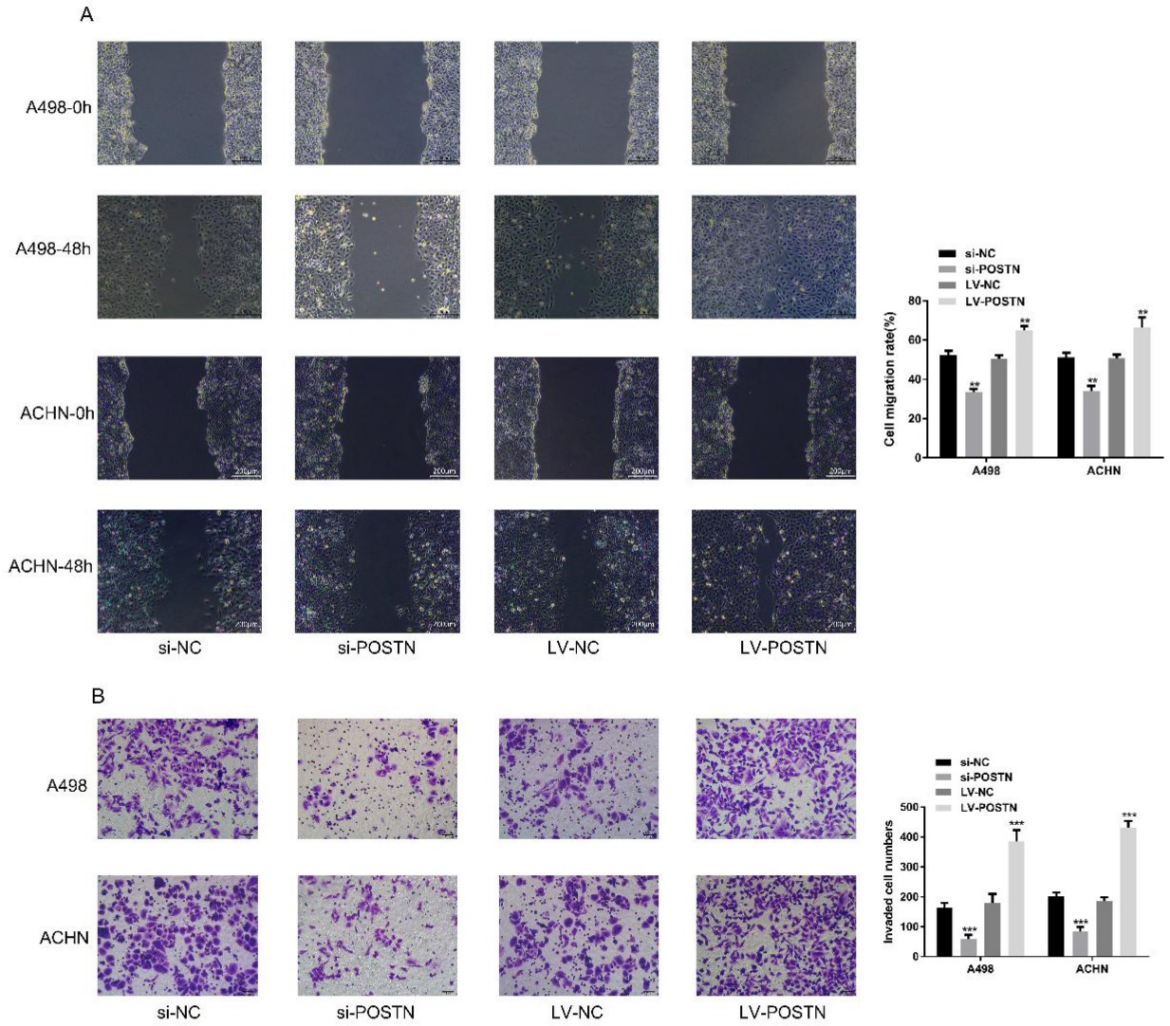
POSTN promotes migration and invasion in RCC cells. (A) Wound-healing assay in ACHN and A498 cells with POSTN knockdown and overexpression (Bar: 200 µm); (B) Migration and invasion assay in ACHN and A498 cells with POSTN knockdown and overexpression (Bar: 50 µm). Data are presented as means ± s.d. **p<0.01, ***p<0.001 as compared with si-NC and LV-NC groups.

In order to figure out the effect of POSTN on migration and invasion in RCC cells, we performed wound healing assay and transwell assay to assess whether POSTN-influenced cell migration and invasion in RCC. Our result demonstrated that the migration and invasion capacity of ACHN and 786-O were significantly weakened after POSTN silencing, while enhanced after overexpressing (Figure 5A, B), which is consistent with previous research^19^ . All these results suggested that POSTN promoted migration and invasion of RCC cells.

### POSTN knockdown promotes A498 and ACHN apoptosis and expression of epithelial‑mesenchymal transition (EMT) markers in RCC

**Figure 6:**
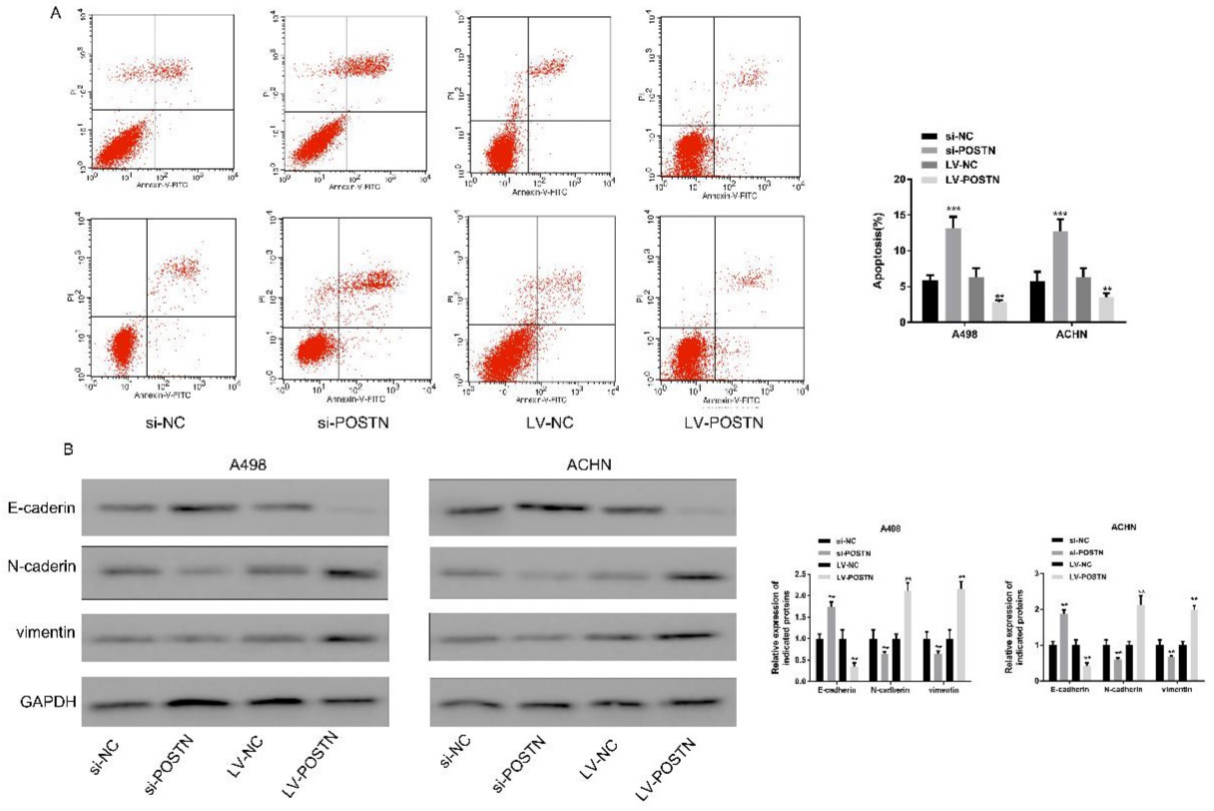
POSTN promoted the apoptosis of RCC cells through EMT. (A) Apoptosis in ACHN and A498 cells detected using flow cytometry after POSTN knockdown and overexpression; (B) western blot analysis for EMT molecular markers in ACHN and A498 cells with POSTN knockdown and overexpression. Data are presented as means ± s.d. **p<0.01, ***p<0.001 as compared with si-NC and LV-NC groups.

Four states, including normal living cells, early apoptotic cells, mid and late apoptotic and necrotic cells and mechanically injured cells, were observed in Annexin V-FITC labels apoptotic cells. In this study, POSTN knockdown significantly increased the RCC cells apoptosis, while decreased after overexpressing (Figure 6A).

EMT plays a critical role in tumor cell development 20. E‑cadherin is widely considered as molecular markers of epithelial cells, and N‑cadherin and vimentin are commonly used for mesenchymal cells recognition 21. As shown in Figure 6B, the expression of E‑cadherin was obviously increased in RCC cells following POSTN knockdown; conversely, the levels of N-cadherin and vimentin were reduced. On the contrary, POSTN overexpression showed opposite tendency of E‑cadherin, N‑cadherin and vimentin expression in RCC cells. All above indicated POSTN may participate in the EMT process in RCC.

### POSTN plays a critical role in the activation of ILK/Akt/mTOR signaling in RCC

**Figure 7:**
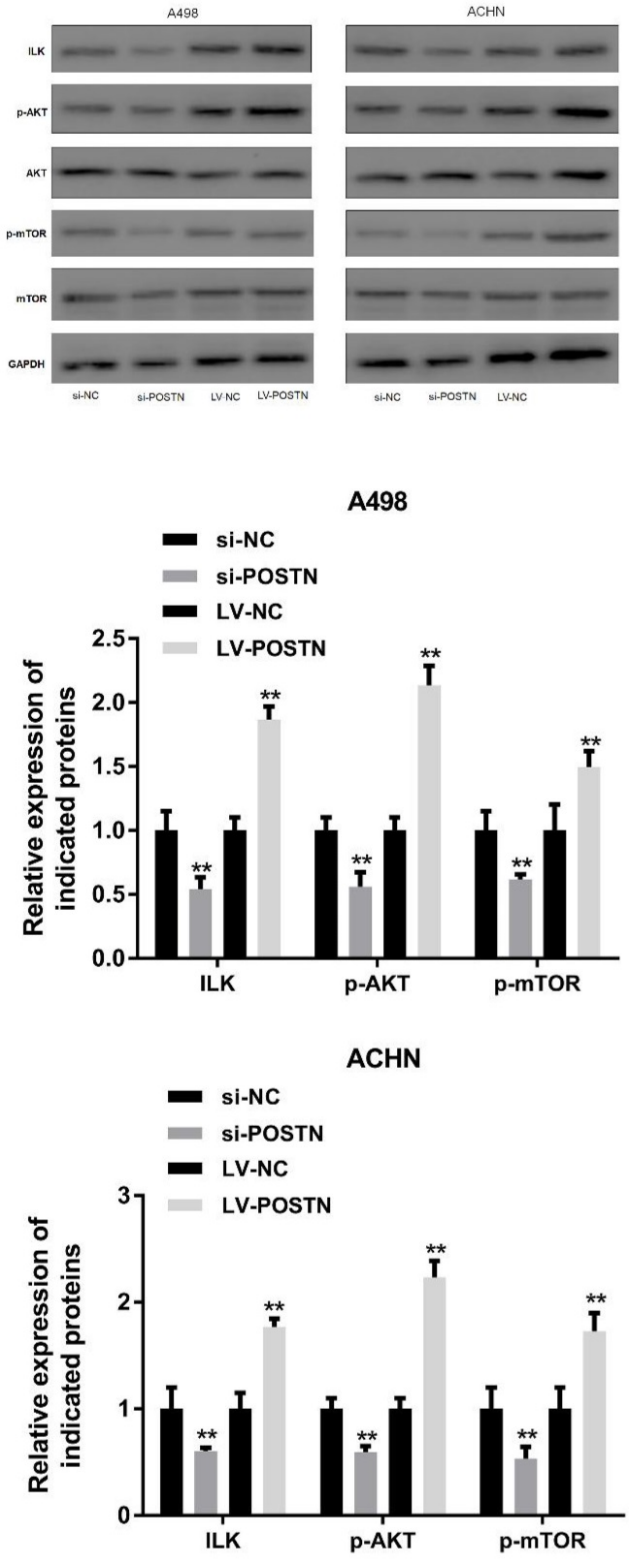
POSTN knockdown and overexpression regulate ILK/AKT/mTOR pathway. **p<0.01 as compared with si-NC and LV-NC groups. Data are presented as means ± s.d. **p<0.01 as compared with si-NC and LV-NC groups.

ILK/AKT and mTOR signaling plays various function in cell development, however, these pathways also involved in human carcinoma progression 22, 23. In the present study, it was demonstrated that the levels of ILK, phosphorylated AKT and mTOR phosphorylation were markedly decreased following POSTN knockdown in A498 and ACHN cells (Figure 7). Conversely, the levels of AKT were not altered, while mTOR expression was notably decreased. On the contrary, upregulated POSTN enhanced levels of ILK, phosphorylated AKT and mTOR phosphorylation in A498 and ACHN cells. These results showed that POSTN promoted cell migration and invasion by activating EMT through ILK /AKT/mTOR pathway.

**Figure 8:**
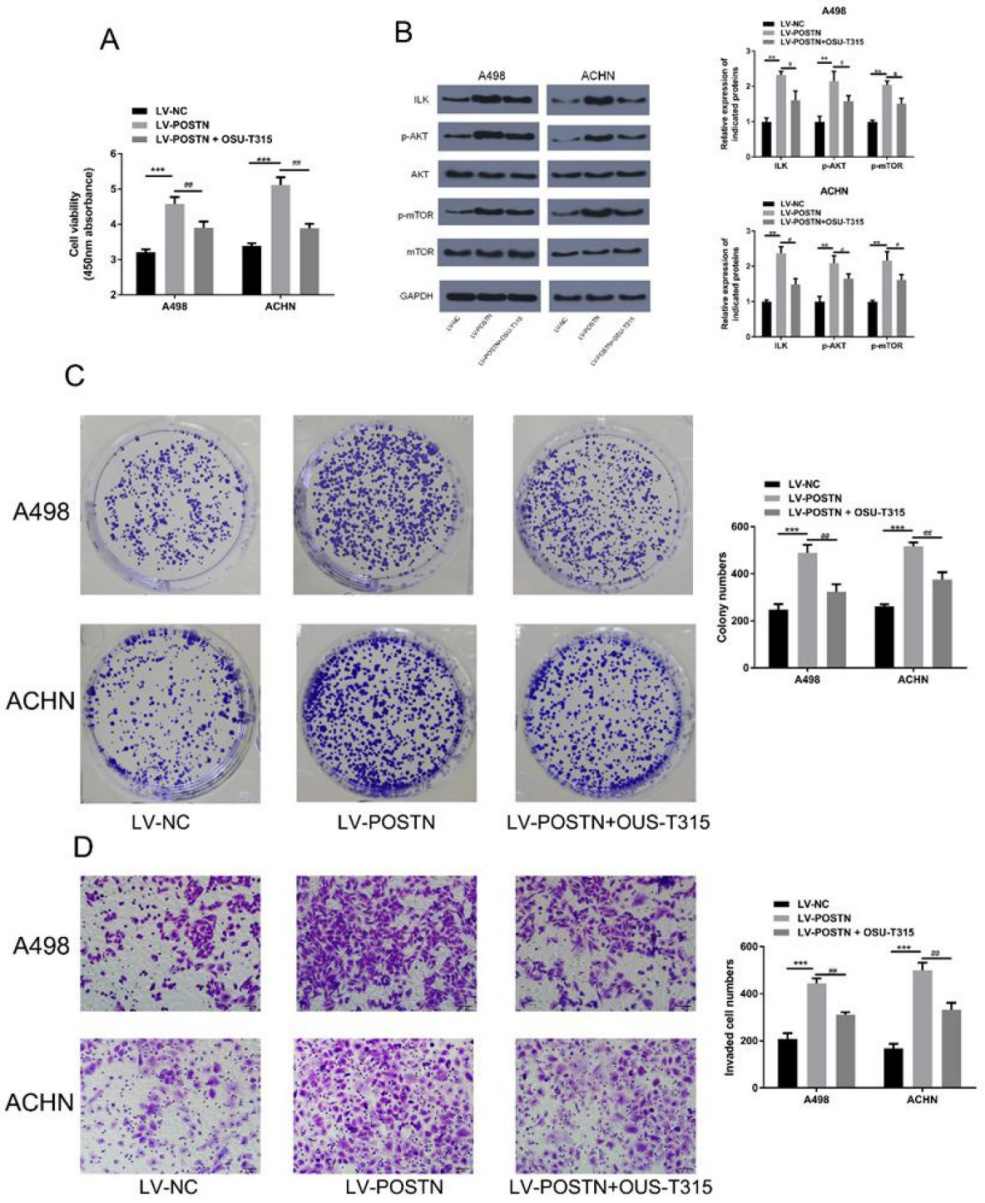
Effect of POSTN overexpression on colonization and invasion in RCC cells after ILK/AKT/mTOR signaling inhibition. (A) ACHN and A498 cells viability assay. (B) Protein expression in ILK/AKT/mTOR pathway. (C) Colony formation after ILK inhibition in ACHN and A498 cells. (D) Invasion assay after ILK inhibition in ACHN and A498 cells (Bar: 50 µm). Data are presented as means ± s.d. **p<0.01, ***p<0.001 as compared with LV-NC group. ^#^p<0.05, ^##^p<0.01 as compared with LV-POSTN group.

To further validate the POSTN role in ILK/Akt/mTOR pathway, we examined the effects of ILK inhibitor (OSU-T315) on POSTN function. In Figure 8A, POSTN overexpression increased the cell viability, while OSU-T315 regulated this trend. In addition, as shown in Figure 8B, POSTN overexpression increased the expression of ILK, whereas OSU-T315 attenuated the increase. Meanwhile, we found the upregulated phosphorylated AKT and mTOR phosphorylation in POSTN overexpression cells were decreased after treatment with OSU-T315. Moreover, we found the increased colony formation capacity and invasion ability of overexpressed POSTN were significantly reversed by OSU-T315 (Figure 8C, D). All these findings proved that POSTN could activated ILK/Akt/mTOR in RCC invasion and colonization.

### Loss of POSTN reduces xenograft tumor growth via ILK /AKT/mTOR pathway

**Figure 9:**
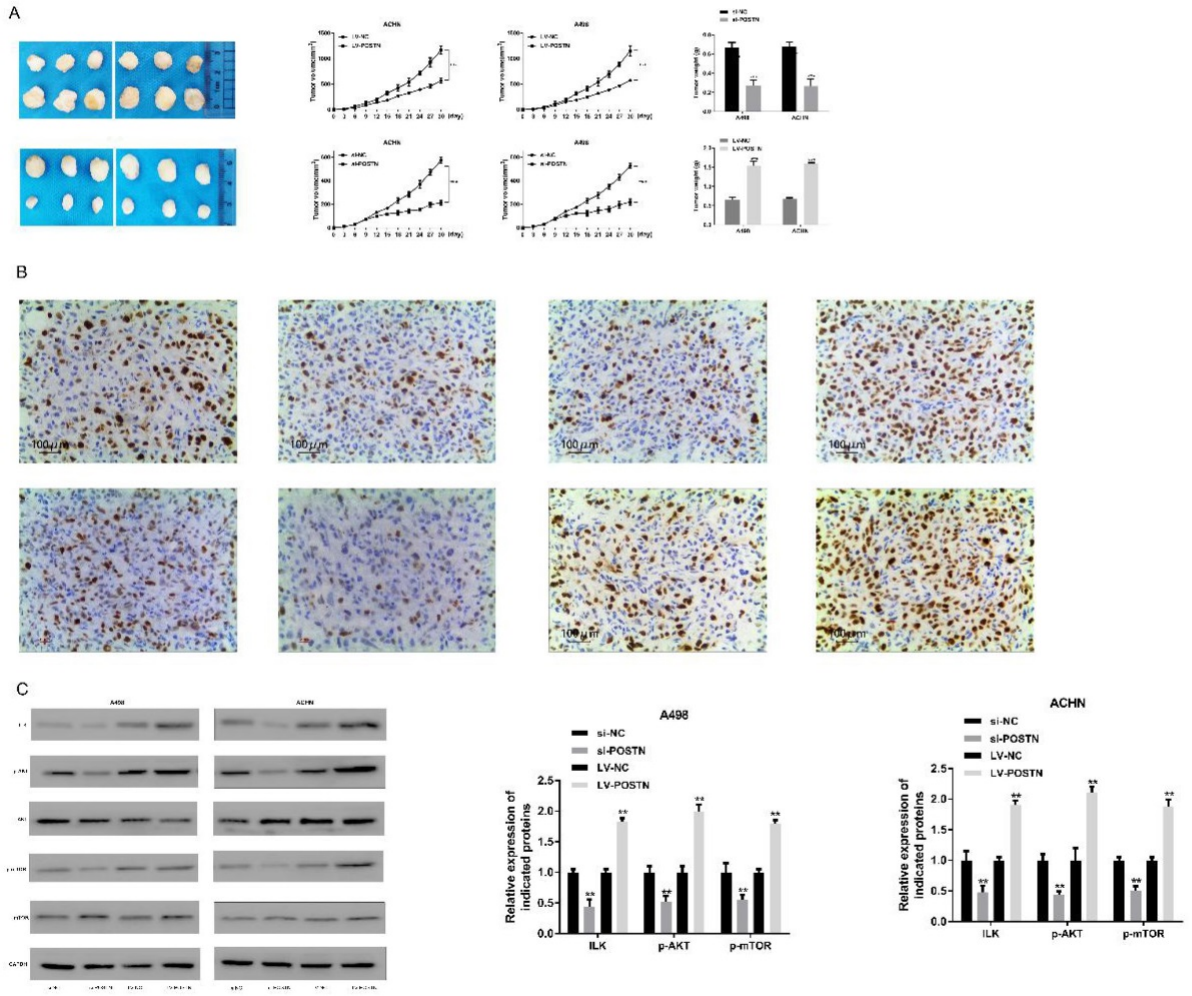
POSTN promotes RCC growth through ILK/AKT/mTOR pathway. (A) growth curves and tumor weights of xenograft tumors generated by subcutaneous injection of POSTN -manipulated ACHN and A498 cells. Data are presented as means ± s.d. *p<0.05, **p<0.01, ***p<0.001 as compared with si-NC and LV-NC groups. (B) Immunohistochemical staining of POSTN in ACHN and A498 xenografted tumors (Bar: 200 µm); (C) Key proteins expressions in ILK/AKT/mTOR pathway in xenograft tumor tissues.

To determine a functional role of POSTN in promoting RCC growth* in vivo*, we utilized a subcutaneous xenograft tumor model implanted with POSTN-manipulated A498 and ACHN cells. Two groups of mice were injected subcutaneously with cells previously transduced with POSTN-knockdown cells, and the other two groups were injected with siRNA scramble. After 15 days, the average volume of tumors derived from A498 and ACHN cells transduced with si-POSTN was significantly reduced, whereas tumors from A498 and ACHN cells receiving si-NC showed continuous growth (Figure 9A), and the size and weight of tumors were approximate 2.4-fold and 3.1-fold increase in si-NC A498 and ACHN cell groups, respectively (Figure 9A). In order to confirm the effect of POSTN in xenograft tumor growth, overexpressed POSTN were also implanted in BALB/c nude mice. As in Figure 9A, LV-POSTN could promoted the size and weight of tumors compared with LV-NC group, which is contrary to the POSTN knockdown groups. In addition, POSTN was decreased in the tumor tissue transduced with POSTN-knockdown cells through IHC analysis (Figure 9B).

On above study, we found POSTN could alter RCC cells growth through ILK/AKT/mTOR pathway. To determine whether this growth factor is involved *in vivo*, western blot was used to analyze the key protein expression in ILK/AKT/mTOR pathway in tumor tissues. As shown Figure 7C, silenced POSTN could significantly decrease the levels of ILK, phosphorylated AKT and mTOR phosphorylation in tumor tissues, while overexpressed POSTN could significantly increase the levels of ILK, phosphorylated AKT and mTOR phosphorylation in xenograft tumor tissues. These results suggested that POSTN promoted tumor growth by activating ILK/AKT/mTOR pathway.

## Discussion

POSTN is an ECM protein, interacted with other ECM proteins or binding to cell integrins, which participates in a variety of biological processes, including tumorigenesis. Previous study suggested POSTN was involved in RCC migration and invasion 18, 24, but the mechanism of between POSTN and cancer metastasis still remain unclear. In this study, we investigated the expression of POSTN in several RCC cells, which POSTN was high-expressed in RCC cells, suggesting POSTN may be an oncogene of renal cell carcinoma. In order to clarify whether POSTN affects the biological behavior of RCC cells, we designed a series of experiments to evaluate the biological function of POSTN. We found that silenced POSTN significantly reduced the proliferation, migration and invasion of RCC cells. On the contrary, overexpressed POSTN enhanced the migration and invasion of RCC cells.

Consistent with previous studies on other cancers, our results showed that POSTN also involved in the movement and invasion of RCC 25-27. These data, combined with a recent report, show that extracellular POSTN can enhance the adhesion of A498 cells, indicating that POSTN plays a key role in the multi-step cascade of cancer metastasis 17. Recent emerged evidence suggested that EMT is a key component of the metastasis process, leading to the separation of connected cells from the primary tumor and intravenous injection into the blood vessels 28, 29. The loss of epithelial markers and the increase of mesenchymal markers are the characteristics of EMT. E-cadherin is an important marker of EMT loss. Vimentin is a cytoskeleton protein, which is not expressed in normal epithelial cells, but widely distributed in the lymphocytes of fibroblasts, endothelial cells and interstitial cells. It has been found that vimentin is also abnormally expressed in a variety of epithelial tumors, which is closely related to the differentiation, invasion and metastasis of cancer cells 30. In this study, our data show that knockout of POSTN can increase the expression of E-cadherin and decrease the expression of N-cadherin. After up-regulating the expression of POSTN, N-cadherin was increased, while the expression of E-cadherin was increased and decreased in RCC cells. These findings suggest that POSTN may be an EMT inducer and may be a new molecular target for anti-metastasis therapy of RCC.

This study confirmed the involvement of POSTN in RCC; however, the mechanism by which POSTN affects the proliferation of RCC cells remains unclear. ILK/AKT is a very important signaling pathway during embryonic development and often has a positive effect on cell growth or proliferation. It has been reported to be overactivated in tumors, with increased phosphorylation of Akt becoming key events 31. In this study, the phosphorylation levels of AKT increased after POSTN overexpression and decreased after POSTN knockdown. These findings suggest that POSTN may regulate ILK/AKT signaling in RCC. mTOR is an important signaling molecule during cell growth, which has been shown to cross-talk with AKT signaling in cancer cells; for example, AKT/mTOR signaling has been reported to play an active role in tumorigenesis of medulloblastoma and thyroid cancer 31, 32. The integrin-linked kinase (ILK) is an intracellular protein serine/threonine kinase that coordinates signaling by integrins and growth factors 33, 34. Number of studies have shown that ILK also has a significant impact on the occurrence and development of human cancer 22, 35, 36. ILK plays a key role in various cell functions related to cell survival, proliferation, exercise, epithelial-mesenchymal transition and angiogenesis 37. Previous study showed that overexpressed ILK during liver oncogenesis was related to AKT activation 22. In this study, POSTN knockdown significantly decrease ILK through AKT inhibition.

In summary, we demonstrated that the role of POSTN on RCC cells, and POSTN could promote migration and invasion in RCC cells. In addition, POSTN triggers EMT process, and activates ILK/Akt/mTOR path way in RCC cells. Therefore, it came to the conclusion that POSTN activated the ILK/AKT/mTOR pathway in proliferation, migration, and invasion process of RCC.
